# Author’s Reply to “A Few Suggestions for Preventing Failure of Ultrasound-Guided Blocks for Below the Shoulder Surgery”

**DOI:** 10.5041/RMMJ.10287

**Published:** 2017-01-30

**Authors:** Anatoli Stav

**Affiliations:** Postanesthesia Care Unit, Hillel Yaffe Medical Center, Hadera, Israel; and The Ruth and Bruce Rappaport Faculty of Medicine, Technion–Israel Institute of Technology, Haifa, Israel

**To the Editor,**

Dr Nair’s letter to the editor regarding the failed nerve blocks mentioned in our paper, “Comparison of the supraclavicular, infraclavicular and axillary approaches for ultrasound-guided brachial plexus block for surgical anesthesia”,^1^ raised several points that I believe are worth looking at in more detail. We are grateful for Dr Nair’s comments which have contributed to the furthering of scholarly discourse.

In general, Dr Nair’s letter relates to the blocks that our research classified as failed. He then discusses various approaches and suggests the reason for the failures of the axillary approach blocks.

## DEFINITION OF BLOCK FAILURE

We had very strong criteria for defining a failed block.^1^ For example, 10 patients (3 from the supraclavicular (SCL) group, 3 from the infraclavicular (ICL) group, and 4 from the axillary (AX) group) received 3–5 mL of lidocaine 1% intra- or subcutaneously before surgery, due to a positive pin-prick test performed by a surgeon. Those patients were absolutely pain-free from the start and up to the end of surgery; nevertheless, their blocks were classified as “failed.” The rationale for their exclusion is related to our desire to obtain maximal comfort for our patients during surgery. Hence, when these patients were evaluated for the loss of sensation, they were asked if they felt a “dull pressure.” These patients answered in the affirmative, and so we chose the path of caution and administered 3–5 mL of 1% lidocaine; as a consequence their blocks were classified as failed.

The surgeons performing the above evaluations used surgical tweezers immediately prior to surgery and were looking for any sensation, including the dull pressure. It is therefore important to point out that 30 min after finishing the block procedure, but before transferring the patients to the operating theatre, the loss of sensation to pin-prick in the area below the shoulder was also examined by an independent anesthesiologist (blinded regarding which brachial plexus block would be used). The independent anesthesiologist used a different pinprick test methodology, as described by Marhofer et al.:^2^ the tip of a 22-G short beveled needle was applied with a force adequate to indent the skin but not enough to puncture it. This action produced a painful sensation on the unblocked side and was compared with the similar procedure in the contralateral (blocked) side. If a consistent pain-free sensation in the “blocked” upper extremity was determined, the patient was transferred to the operating theatre. There, the surgeon performed a second pin-prick test, as described above, using surgical tweezers. The difference between the dull but not painful feeling in the surgeon’s test compared to the test response produced by the independent anesthesiologist is key to understanding the classification of those 10 blocks as “failed.”^1^

There is some controversy as whether or not the 10 blocks should have been interpreted as failed at all; however, we wanted to be as strict and accurate as possible. The opinion of my co-author, L. Reytman, 1 is that all 10 “failed” blocks should have been classified as “appropriate.”

## THE NERVE BLOCK FOR THE SCL APPROACH

Dr Nair wrote: “In the SCL approach … the ulnar nerve is usually spared as the inferior trunk is located medially. A medially directed needle is not recommended as there is a high risk of pneumothorax due to inadvertent puncture of the pleura.”

Our SCL block was performed according to the Jack Vander Beek technique,^3^ after identifying the brachial plexus just posterior to the clavicle (low Fowler position with the head turned contralaterally), superior and lateral to the subclavian artery. The needle was inserted in-plane from the posterolateral edge of the ultrasound (US) probe into the sheath (but not into the fascicles, which could be seen as hypoechoic structures into the sheath of the brachial plexus) containing the nerves of the brachial plexus. Normal saline (1–2 mL) was injected after an aspiration test for additional confirmation of needle tip placement, followed by slow injection of 30 mL of local anesthetic (LA). After repositioning needle into the “corner pocket” (the angle between the subclavian artery and the first rib on the posterolateral side) the last 10 mL of LA was injected for appropriate blockade of the ulnar nerve.

We agree with Soares et al.^4^ that the optimal needle position for a successful SCL block of the ulnar nerve is a “corner pocket;” however, we do not agree with Dr Nair’s recommendation that “… for a 100% successful block the anesthesiologist should block the ulnar nerve separately using the SCL approach …” We have been using the SCL block for more than 5.5 years with a very high success rate; we have never needed to perform an additional separate ulnar nerve block.

## FAILED BLOCKS IN THE AX GROUP

Two patients in our AX group received sedation (fentanyl 50–150 μg IV with midazolam 3–5 mg IV), but the operation was completed without using general anesthesia. We classified both blocks as “failed.” This point may also be controversial. The doses of fentanyl and midazolam did not produce general anesthesia, only moderate sedation.

It is true that “one patient from the AX group felt pain in the area innervated by the ulnar nerve; general anesthesia was therefore used.”^1^ A separate additional block of the ulnar nerve was not considered for this patient due to the following considerations:

- The surgical procedure had already been started. To perform an additional ulnar block would have necessitated postponing the operation for at least 45 min (15 min to prepare for and perform the block and 30 min for LA to affect the target nerve). This, in our opinion, would have been a poor choice as compared to giving general anesthesia and performing the procedure without delay.
- All patients received LA of 40 mL bupivacaine 0.5% with adrenaline 1:200000 for the block (see additional information below regarding the ultrasound-guided block techniques that we use). An additional LA injection might have exceeded the permissible dose, resulting in LA toxicity. Therefore, we believe that, for this particular case, the correct decision was made to switch to general anesthesia.

## ICL APPROACH FOR ELBOW SURGERIES

Dr Nair wrote: “ICL approach ... is the best approach for elbow surgeries.”

We concluded in our present work^1^ that “US-guided brachial plexus block can be performed using the three approaches, supra- or infraclavicular or axillary, with a similar quality of surgical anesthesia for operations of the upper extremity below the shoulder.” We do not see in Dr Nair’s letter any data that might disprove our conclusion. Therefore such a statement should be considered unproven and subjective. It is inappropriate to recommend this statement to anyone else without the appropriate supporting data.

## ICL APPROACH FOR CATHETER PLACEMENT FOR CONTINUOUS ANALGESIA

Dr Nair cited the work of Sandhu and Capan:^5^ “This [ICL] approach is best suited for catheter placement for continuous analgesia.”

The above statement may be true; however, it was not a variable that was analyzed for our research. Furthermore, in our practice we use a continuous brachial plexus block via all three approaches (SCL, ICL, and AX) after below-the-shoulder operations (for example for treatment of continuous passive motion after elbow adhesiolysis). To date, the rate of catheter dislodgment has been identical in all groups. However, only a small number of patients have been treated by those techniques, and it is impossible at this point to make any conclusion regarding the preferred of the three possible approaches.

## AX APPROACH FOR HAND SURGERIES

Dr Nair cited the textbook of Hadzic^6^ and wrote: “The AX approach is ideal for hand surgeries and has the least complications. Musculocutaneous nerve sparing is the most common problem with AX block, particularly when using nerve stimulation and landmark techniques.”

We agree with this statement. Prior to the era of US-guided regional anesthesia when landmark or nerve stimulation techniques were used, SCL and ICL approaches were rarely employed due to the high complication rate and failed results. Blocking of the musculocutaneous nerve (AX approach) was difficult and had a high failure rate. The US-guided technique was the beginning of a revolution in relation to peripheral nerve blocks. Regional anesthesia, and especially peripheral nerve blockade, ceased to be an art—it is now a science accessible to everyone. With regard to the US-guided technique of musculocutaneous nerve (MCN) block, please see additional information at the end of this response.

Dr Nair cited the work of Schafhalter-Zoppoth and Gray.^7^ These authors published an investigation conducted on 19 volunteers for assessment of the anatomical variants of the MCN near the coracobrachialis muscle in the axilla, describing those variants with a corresponding US picture. The authors note in particular that in two cases the MCN could not be identified with the help of US.

Loukas and Aqueelah published the results of 129 dissections and proposed a classification of the possible variation of connections between median and musculocutaneous nerves in relation to the point of entry of the MCN into the coracobrachialis muscle.^8^ Guerri-Guttenberg and Ingolotti proposed a classification of the MCN variations.^9^ The authors not only found a difference in the anatomy of the communication point between the median and musculocutaneous nerves, but also the anatomy of the points of penetration of the coracobrachialis muscle by the MCN. In 6 cases out of 54 dissections, the MCN did not penetrate the coracobrachialis muscle at all. In two cases the MCN was absent.^9^

We experienced no difficulties in identifying the MCN by US in our study.^1^ In several cases we identified this nerve between the biceps and the coracobrachialis muscles; in other cases we identified the nerve in the mass of the coracobrachialis muscle. In everyday practice we do indeed come across isolated cases where detection of the MCN is impossible by US. For those cases, we prefer to block the brachial plexus using different approaches, for example the SCL approach.

## ADDITIONAL INFORMATION ABOUT THE ULTRASOUND-GUIDED BLOCK TECHNIQUES USED IN OUR STUDY

All brachial plexus blocks were performed using a LA of 40 mL of bupivacaine 0.5% with adrenaline 1:200000.

The SCL block was carried out according to the Jack Vander Beek technique,^3^ as discussed above.

The ICL block was carried out according to the modified Sandhu and Capan technique.^5^ With the patient in the supine position, the arm was abducted to 90°, and the head was turned toward the contralateral side. The US probe was located 2 cm medial and inferior to the coracoid process. After identifying the three cords of the brachial plexus (BP) around the axillary artery posterior to the pectoralis minor muscle, a Pajunk 21-G 80 mm or 100 mm needle (not the Tuohy needle used by Sandhu and Capan^5^) was inserted and placed between the axillary artery and vein (directed toward the medial cord), followed by hydrodissection with 5–6 mL normal saline. If the saline spread around the artery, 40 mL of LA were injected slowly. If the injected fluid was not seen to spread around the artery, the needle was reinserted and the injection was repeated. In many cases relocation of the needle was needed for separate blockade of medial, lateral, and posterior cords of the BP.

The AX block was carried out according to the Jack Vander Beek technique,^10^ with the patient in a supine position, arm abducted 90°, elbow flexed 90°, and the palm resting next to the head. The US probe was placed transverse to the axillary artery. After identification of the median, ulnar, and radial nerves around the axillary artery in the axillary space, the needle was inserted by in-plane approach and LA was injected around each of the nerves after a negative aspiration test. The probe was moved distally for visualization and blocking of the MCN. The MCN can usually be well visualized between the biceps and coracobrachialis muscles or in the mass of the coracobrachialis muscle. We blocked this nerve with a separate injection of LA as described by Hadzic.^11^ The total volume of injected LA was 40 mL.

Additional nerves were blocked by subcutaneous local infiltration with 10 mL of 1% lidocaine in the axillary space. A hemi-ring injection of lidocaine 1% was used in all patients to eliminate tourniquet pain and pain in the area of distribution of the inter-costobrachial (Th2) and medial brachial cutaneous (Th1 and Th2) nerves.^11^,^12^

## Abbreviations

AX: axillary
BP: brachial plexus
ICL: infraclavicular
LA: local anesthetic
MCN: musculocutaneous nerve
SCL: supraclavicular
US: ultrasound

## References

[1] Stav A, Reytman L, Stav M-Y (2016). Comparison of the supraclavicular, infraclavicular and axillary approaches for ultrasound-guided brachial plexus block for surgical anesthesia. Rambam Maimonides Med J.

[2] Marhofer D, Karmakar MK, Marhofer P, Kettner SC, Weber M, Zeitlinger M (2014). Does circumferential spread of local anaesthetic improve the success of peripheral nerve block?. Br J Anaesth.

[3] Vander Beek J, Vander Beek J (2009). Supraclavicular Brachial Plexus Block. The Neuraxiom Playbook of 9 Essential Blocks: A Handbook of Ultrasound Guided Regional Nerve Blocks.

[4] Soares LG, Brull R, Lai J, Chan VW (2007). Eight ball, corner pocket: the optimal needle position for ultrasound-guided supraclavicular block. Reg Anesth Pain Med.

[5] Sandhu NS, Capan LM (2002). Ultrasound-guided infraclavicular brachial plexus block. Br J Anaesth.

[6] Hadzic A (2006). Textbook of Regional Anesthesia and Acute Pain Management.

[7] Schafhalter-Zoppoth I, Gray AT (2005). The musculocutaneous nerve: ultrasound appearance for peripheral nerve block. Reg Anesth Pain Med.

[8] Loukas M, Aqueelah H (2005). Musculocutaneous and median nerve connections within, proximal and distal to the coracobrachialis muscle. Folia Morphol (Warsz).

[9] Guerri-Guttenberg RA, Ingolotti M (2009). Classifying musculocutaneous nerve variations. Clin Anat.

[10] Vander Beek J, Vander Beek J (2009). Axillary Brachial Plexus Block. The Neuraxiom Playbook of 9 Essential Blocks: A Handbook of Ultrasound Guided Regional Nerve Blocks.

[11] Hadzic A, Hadzic A (2012). Ultrasound-guided Axillary Brachial Plexus Block. Hadzic’s Peripheral Nerve Blocks and Anatomy for Ultrasound-Guided Regional Anesthesia.

[12] Moore DC, Moore DC (1981). Supraclavicular Approach for Block of the Brachial Plexus. Regional Block: A Handbook for Use in the Clinical Practice of Medicine and Surgery.

